# Supplementary material for: Systems approach identifies monocyte imbalance in symptomatic and asymptomatic P. vivax malaria
Source: Mol Syst Biol. 2025 Aug 19;21(11):1434–65. doi: 10.1038/s44320-025-00135-z (PMC12583509; doi:10.1038/s44320-025-00135-z)
Supplement: Supplementary file 1 — Table EV1 [file 44320_2025_135_MOESM1_ESM.docx]

**Table EV1. Rotational gene set enrichment testing with selected modules against symptomatic *P. vivax* malaria vs healthy community controls.**

PropDown: proportion of genes downregulated the gene set. PropUp = proportion of genes upregulated in the gene set. Direction: direction of change for genes in test set against background set. FDR: two-sided directional false discovery rate. Significance was tested using the mroast test in limma.

| **Module** | **PropDown** | **PropUp** | **Direction** | **FDR** |
| --- | --- | --- | --- | --- |
| Black | 0.001 | 0.742 | Up | < 0.001 |
| Greenyellow | 0.000 | 0.814 | Up | < 0.001 |
| Grey60 | 0.502 | 0.035 | Down | 0.066 |
| Pink | 0.560 | 0.040 | Down | < 0.001 |
| Salmon | 0.710 | 0.008 | Down | < 0.001 |
| Tan | 0.107 | 0.266 | Up | 0.028 |
