# Supplementary material for: Systems approach identifies monocyte imbalance in symptomatic and asymptomatic P. vivax malaria
Source: Mol Syst Biol. 2025 Aug 19;21(11):1434–65. doi: 10.1038/s44320-025-00135-z (PMC12583509; doi:10.1038/s44320-025-00135-z)
Supplement: Supplementary file 2 — Table EV2 [file 44320_2025_135_MOESM2_ESM.docx]

**Table EV2. Rotational gene set enrichment testing with selected modules against symptomatic vs asymptomatic *P. vivax* malaria.**

PropDown: proportion of genes downregulated the gene set. PropUp = proportion of genes upregulated in the gene set. Direction: direction of change for genes in test set against background set. FDR: two-sided directional false discovery rate. Significance was tested using the mroast test in limma.

| Module | PropDown | PropUp | Direction | FDR |
| --- | --- | --- | --- | --- |
| Black | 0.001 | 0.780 | Up | < 0.001 |
| Greenyellow | 0.009 | 0.593 | Up | 0.006 |
| Grey60 | 0.095 | 0.082 | Up | 0.958 |
| Pink | 0.267 | 0.117 | Down | 0.033 |
| Salmon | 0.639 | 0.002 | Down | < 0.001 |
| Tan | 0.339 | 0.078 | Down | 0.001 |
