# Supplementary material for: Systems approach identifies monocyte imbalance in symptomatic and asymptomatic P. vivax malaria
Source: Mol Syst Biol. 2025 Aug 19;21(11):1434–65. doi: 10.1038/s44320-025-00135-z (PMC12583509; doi:10.1038/s44320-025-00135-z)
Supplement: Supplementary file 5 — Expanded View Figures [file 44320_2025_135_MOESM5_ESM.pdf]

Expanded View Figures

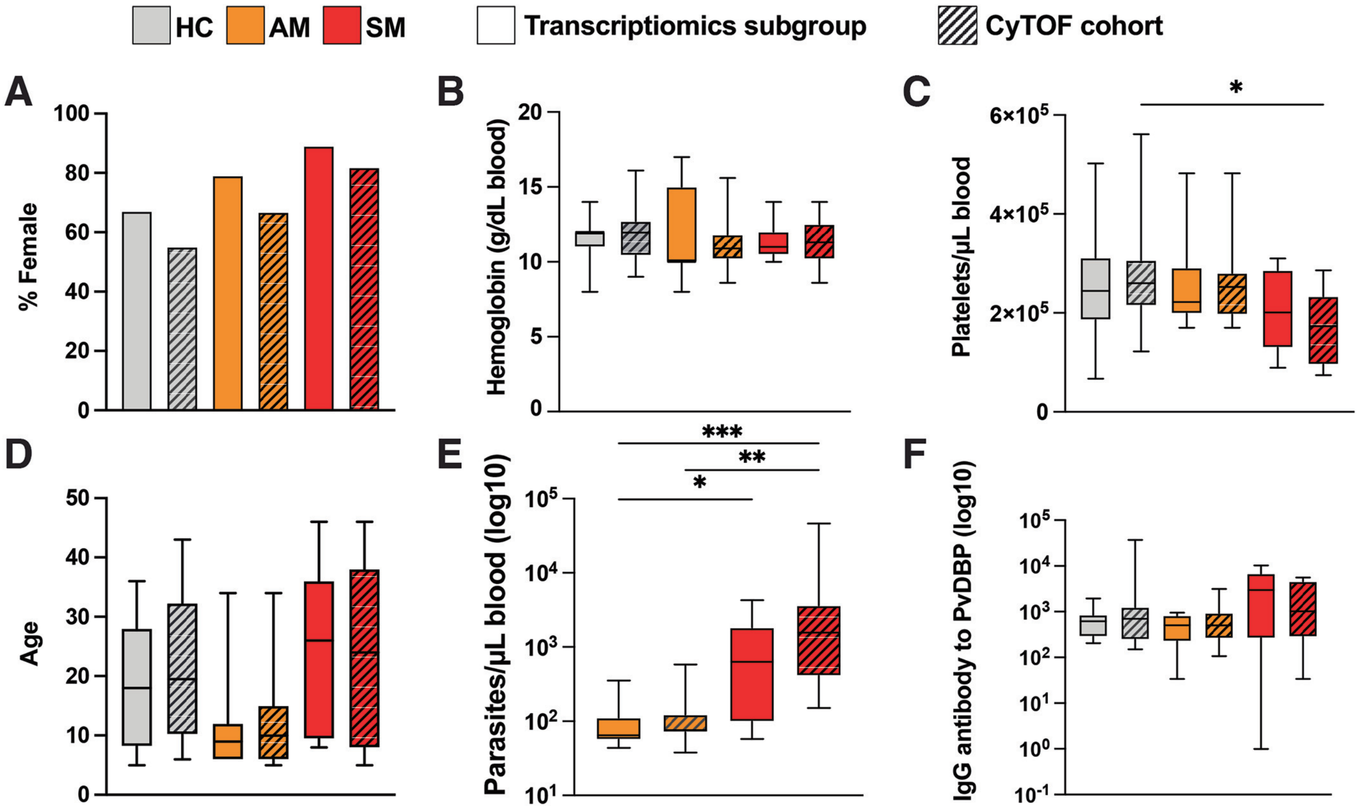

Figure EV1. CyTOF cohort and transcriptomics sub-cohort characteristics.

(**A–F**) Immune phenotyping by CyTOF was conducted with *P. vivax* symptomatic ($n = 11$ SM) and *P. vivax* asymptomatic ($n = 19$ AM) infected participants as well as *P. vivax* negative healthy community controls ($n = 24$ HC) (striped bars). Transcriptional profiling was conducted with a sub-cohort of *P. vivax* symptomatic ($n = 9$) and *P. vivax* asymptomatic ($n = 11$) infected participants, as well as *P. vivax* negative healthy immune control ($n = 12$) (full bars). Clinical parameters determined in the study include gender (**A**), hemoglobin (g/dL blood) (**B**), platelets/μL blood (*$P = 0.0372$) (**C**), age (**D**), parasite density (*$P = 0.0177$, **$P = 0.0011$, ***$P = 0.0002$) (**E**), and IgG antibody to *P. vivax* Duffy binding protein (**F**). Boxes represent the 25th to 75th percentile, whiskers show the range (minimum to maximum), and lines represent the median. Significance was determined by the Chi-square test (**A**) and the Kruskal–Wallis test with Dunn's multiple comparisons (**B–F**).

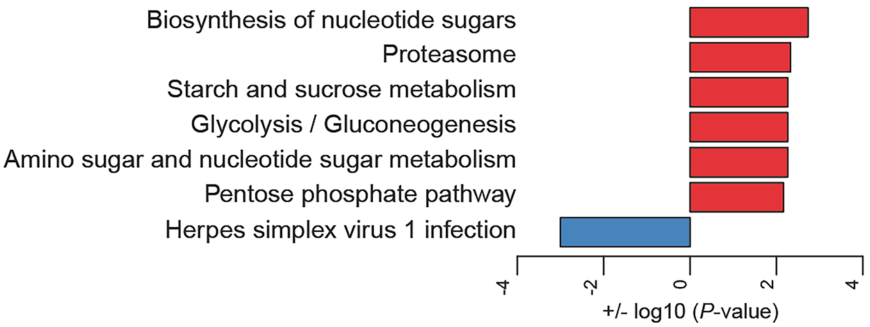

Figure EV2. Kyoto Encyclopedia of Genes and Genomes (KEGG) pathway analysis was conducted for genes DE between symptomatic and asymptomatic *P. vivax* malaria.

Gene expression profiles of PBMCs from symptomatic ($n = 9$) and asymptomatic ($n = 11$) *P. vivax*-infected individuals were compared. Bar plots showing significantly enriched KEGG pathways identified using hypergeometric testing implemented with the kegga function in limma and scaled by + or − log10 (*P* value). Red KEGG pathways are enriched in symptomatic malaria and blue KEGG pathways are enriched in asymptomatic malaria.

 © The Author(s)

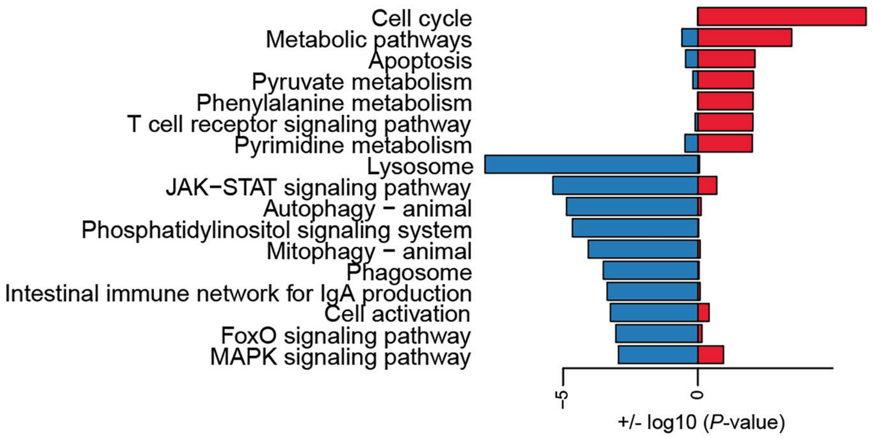

Figure EV3. Kyoto Encyclopedia of Genes and Genomes (KEGG) pathway analysis was conducted for genes DE between symptomatic *P. vivax* malaria and healthy community controls.

Gene expression profiles of PBMCs from symptomatic ($n = 9$) *P. vivax*-infected individuals and healthy immune controls ($n = 12$) were compared. Bar plots showing significantly enriched KEGG pathways identified using hypergeometric testing implemented with the kegga function in limma and scaled by + or −log10 (*P* value). Red KEGG pathways are upregulated and blue KEGG pathways are downregulated in symptomatic malaria.

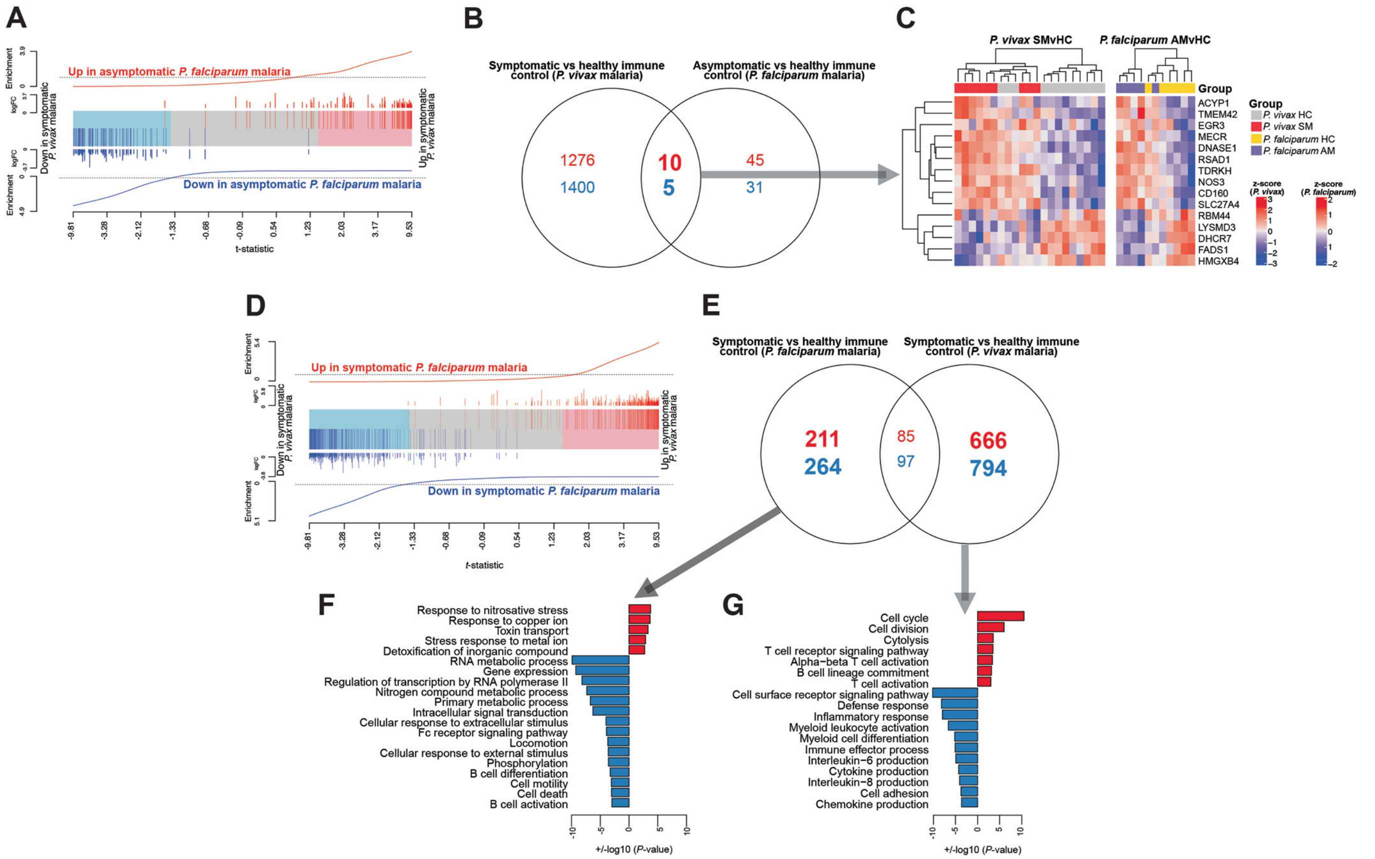

Figure EV4. Symptomatic *P. vivax* malaria induces immunoregulatory transcripts and a reduced inflammatory response than symptomatic *P. falciparum* malaria.

Gene expression profiles between symptomatic *P. vivax* malaria and healthy immune controls were compared with asymptomatic and symptomatic *P. falciparum* malaria and healthy immune controls (Studniberg et al, 2022). (**A**) Barcode plot showing significant positive correlation ($P < 0.0001$) between genes regulated in asymptomatic *P. falciparum* malaria vs healthy immune controls against genes regulated in symptomatic *P. vivax* malaria vs healthy immune controls. Significance was tested using the roast test in limma. (**B**) Venn diagram showing the overlap of differentially expressed genes between symptomatic *P. vivax* vs healthy immune controls, and asymptomatic *P. falciparum* vs healthy immune controls. A total of 15 genes were co-regulated by both symptomatic *P. vivax* and asymptomatic *P. falciparum* malaria. (**C**) Heatmap showing expression of the 15 genes co-regulated between symptomatic *P. vivax* and asymptomatic *P. falciparum* malaria identified in (**B**) against healthy immune controls in the *P. vivax* study on the left, and the *P. falciparum* study on the right. (**D**) Barcode plot showing significant positive correlation ($P < 0.0001$) between genes regulated in symptomatic *P. falciparum* malaria vs healthy immune controls against genes regulated in symptomatic *P. vivax* malaria vs healthy immune controls. Significance was tested using the roast test in limma. (**E**) Venn diagram showing the overlap of differentially expressed genes between symptomatic *P. vivax* vs healthy immune controls, and symptomatic *P. falciparum* vs healthy immune controls. A total of 1460 genes were uniquely regulated by symptomatic *P. vivax* malaria, and a total of 475 genes were uniquely regulated by symptomatic *P. falciparum* malaria. (**F**, **G**) Bar plots showing significantly enriched gene ontology (GO) terms scaled by $+/-$ log10 (P value) in genes uniquely regulated by symptomatic *P. vivax* malaria (**F**) and genes uniquely regulated by symptomatic *P. falciparum* malaria (**G**) identified using hypergeometric testing implemented with the goana function in limma and scaled by $+$ or $-$log10 (P value). Red GO terms are upregulated, and blue GO terms are downregulated in symptomatic malaria against healthy immune controls.

 © The Author(s)

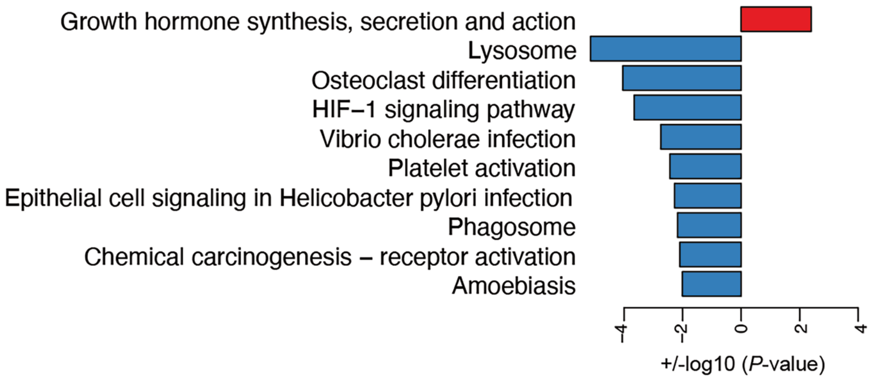

Figure EV5. Kyoto Encyclopedia of Genes and Genomes (KEGG) pathway analysis was conducted for genes DE between asymptomatic *P. vivax* malaria and healthy community controls.

Gene expression profiles of PBMCs from asymptomatic ($n = 11$) *P. vivax*-infected individuals and healthy immune controls ($n = 12$) were compared. Bar plots showing significantly enriched KEGG pathways identified using hypergeometric testing implemented with the kegga function in limma and scaled by + or −log10 (*P* value). Red KEGG pathways are upregulated and blue KEGG pathways are downregulated in asymptomatic malaria.

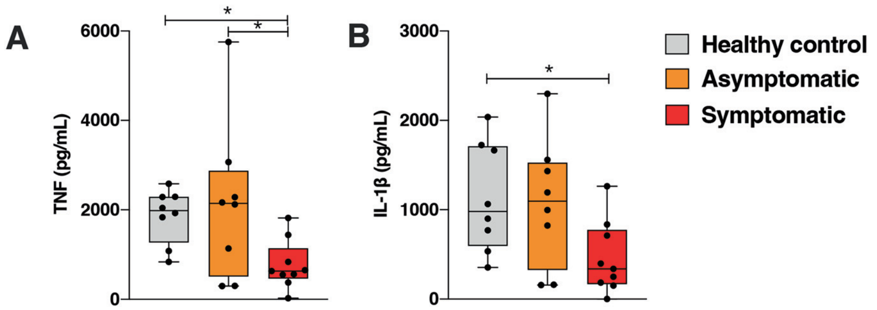

Figure EV6. Reduced cytokine responses in monocytes from *P. vivax*-infected individuals relative to healthy immune controls.

Blood monocytes were isolated from PBMCs of *P. vivax* symptomatic ($n = 8$) and *P. vivax* asymptomatic ($n = 8$) infected participants, as well as *P. vivax* negative healthy immune controls ($n = 8$). Cells were then plated at a density of 1×10^5 cell/ml and stimulated with LPS (50 ng/ml) for 24 h. (**A**, **B**) Cell culture supernatants were harvested and their TNF (**A**) and IL-1β (**B**) levels were measured by capture ELISA. Boxes represent the 25th to 75th percentile, whiskers show the range (minimum to maximum), and lines represent the median. Significance was determined by Kruskal–Wallis test with Dunn's multiple testing. $^*P = 0.0101$ for TNF values between symptomatic individuals and healthy controls, $^*P = 0.0288$ for TNF values between symptomatic individuals and asymptomatic individuals, $^*P = 0.0226$ for IL-1β values between symptomatic individuals and healthy controls.

 © The Author(s)

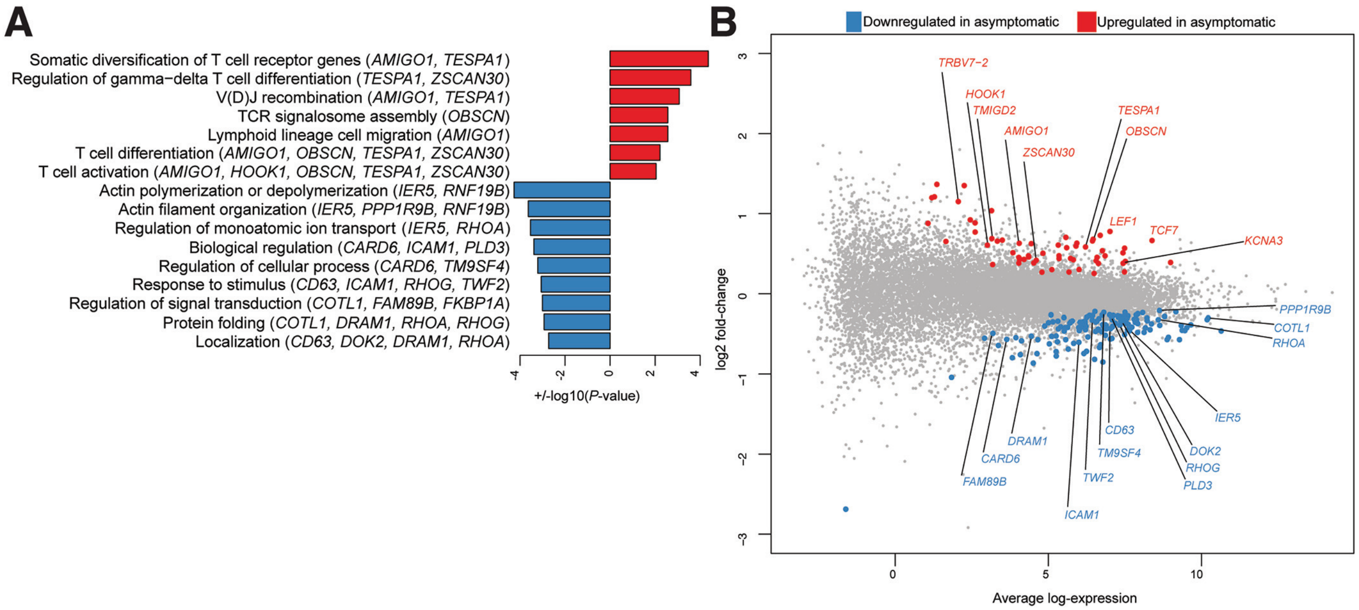

Figure EV7. ***P. vivax* asymptomatic malaria is permissive of normal T-cell differentiation but compromises immune effector function.**

Gene expression profiles for 83 genes uniquely regulated by *P. vivax* asymptomatic (AM) infected participants and healthy immune controls (HC). (**A**) Bar plots showing significantly enriched gene ontology (GO) terms identified using hypergeometric testing implemented with the goana function in limma and scaled by + or − log10 (P value). Red GO terms are upregulated, and blue GO terms are downregulated in AM. (**B**) Mean-difference plot displaying DEGs between AMvHC. Each gene is plotted as a single point determined by Log2-fold change and average transcript abundance. Red genes are overrepresented, and blue genes are underrepresented in AM.

© The Author(s)

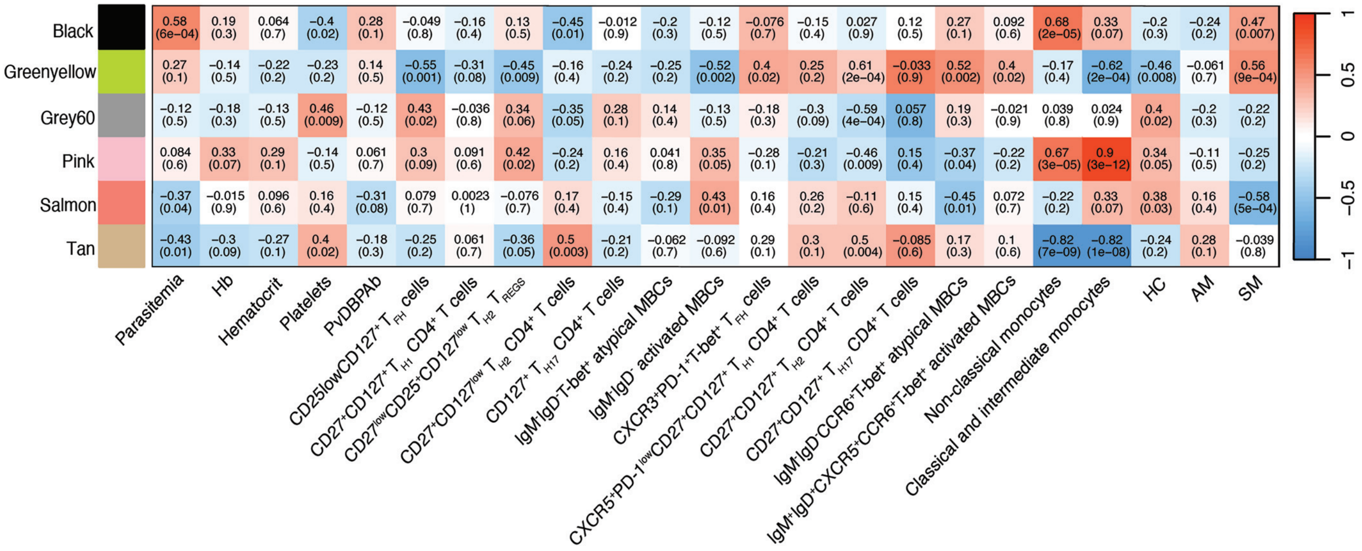

Figure EV8. Weighted gene co-expression network analysis of the immune response to *P. vivax* malaria.

Co-expression network analysis with whole-genome expression profiles of peripheral blood mononuclear cells from *P. vivax* symptomatic (SM, $n = 9$), asymptomatic (AM, $n = 11$) and healthy control (HC, $n = 12$) individuals. Expanded correlation heatmap from Fig. 6B, depicting associations between gene co-expression modules significantly enriched for at least one pairwise contrast in the limma-voom differential expression analysis, clinical traits, cell populations associated with increased or reduced odds of *P. vivax* malaria (Ioannidis et al, 2021), monocyte frequencies estimated by cell-type deconvolution and clinical groups. Red represents positive correlations and blue represents negative correlations.

© The Author(s)

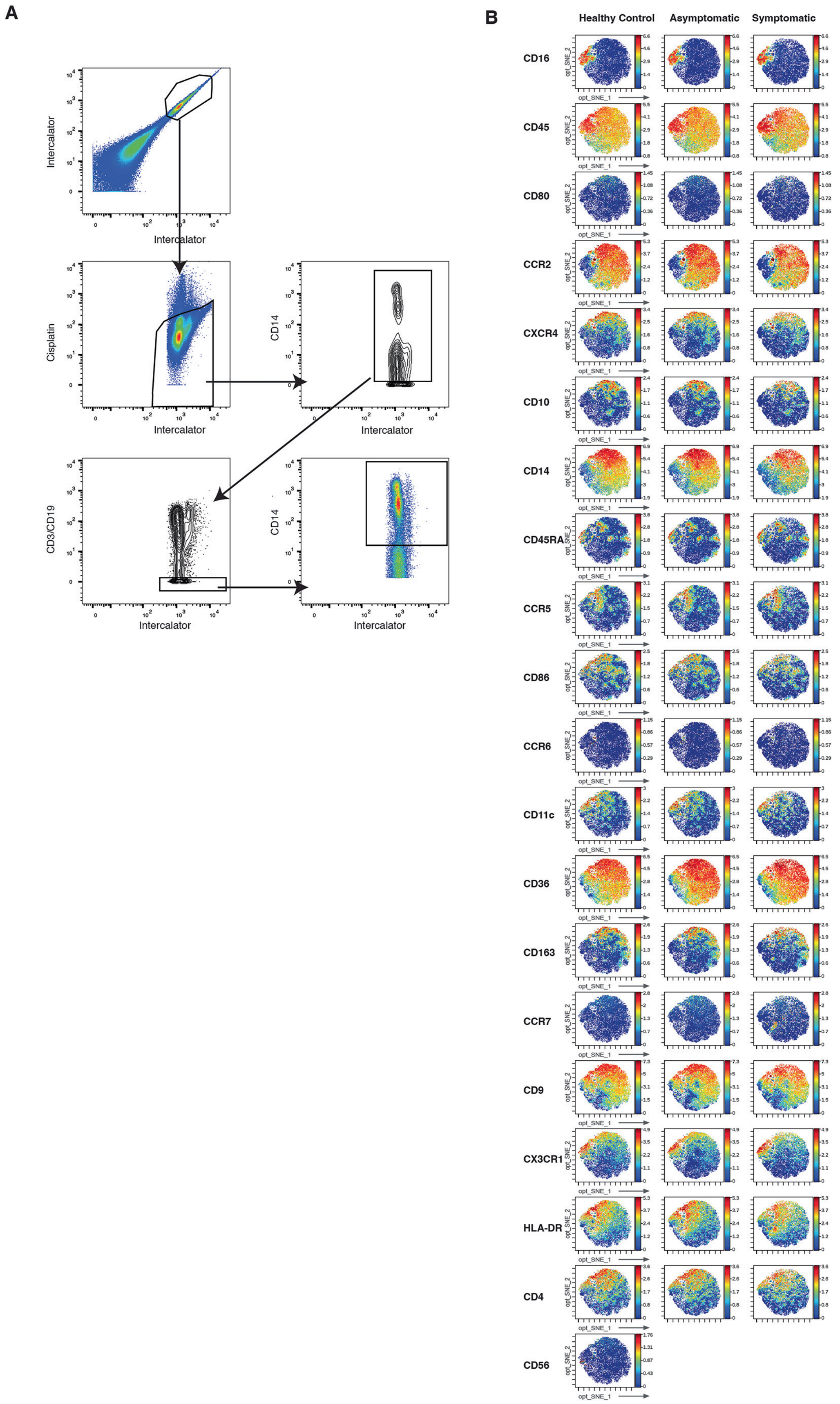
A
Intercalator
Cisplatin
CD14
CD3/CD19
B
Healthy Control
Asymptomatic
Symptomatic
CD16
CD45
CD80
CCR2
CXCR4
CD10
CD14
CD45RA
CCR5
CD86
CCR6
CD11c
CD36
CD163
CCR7
CD9
CX3CR1
HLA-DR
CD4
CD56
opt_SNE_1
opt_SNE_2

Figure EV9. Gating strategy for analysis of blood monocytes by CyTOF.

Peripheral blood mononuclear cells from *P. vivax* symptomatic ($n = 8$), asymptomatic ($n = 8$), and healthy controls ($n = 8$) were stained with a panel of metal-labeled antibodies and analyzed CyTOF. (**A**) Manual gating was used to select for $CD14^+$ monocytes. (**B**) vi-SNE analysis was performed on pooled monocytes for each group and the cell surface expression was illustrated by their density.

 © The Author(s)
